# Joining of SiC Ceramic by Si–C Reaction Bonding Using Organic Resin as Carbon Precursor

**DOI:** 10.3390/ma15124242

**Published:** 2022-06-15

**Authors:** Xishi Wu, Qing Huang, Yunzhou Zhu, Zhengren Huang

**Affiliations:** 1 Engineering Laboratory of Advanced Energy Materials, Ningbo Institute of Materials Technology & Engineering, Chinese Academy of Sciences, Ningbo 315201, China; wuxishi@nimte.ac.cn (X.W.); huangqing@nimte.ac.cn (Q.H.); 2 State Key Laboratory of High Performance Ceramics and Superfine Microstructure, Shanghai Institute of Ceramics, Chinese Academy of Sciences, Shanghai 201800, China

**Keywords:** SiC ceramic, joining technology, MgCl_2_, phenolic resin/alcohol carbon precursor, grains and interfaces

## Abstract

In this study, the joining of silicon carbide (SiC) ceramics was achieved via a Si–C reaction bonding method using the phenolic resin (PF)–MgCl_2_ system as the carbon precursor. Specifically, by adding MgCl_2_ to the phenolic resin mixture, the average pore size of the product of carbonization of the PF resin mixture increased from 14 ± 5 nm to 524 ± 21 nm, which was beneficial for the infiltration of molten silicon at high temperature. The microstructure of the joined specimens and the effect of the inert filler on the joint strength were investigated. It was demonstrated that SiC–SiC joints with strong interfacial bonding and high flexural strength could be obtained by the Si–C reaction bonding method using a phenol formaldehyde resin/alcohol sol-gel system as the carbon precursor. The flexural strength of the joined specimens reached the highest value, i.e., 308 ± 27 MPa when the solid loading of the inert filler was 26%. Overall, stable joining of silicon carbide ceramics was achieved by the proposed method, which has significance for realizing the preparation of complex-shaped or large silicon carbide ceramic parts.

## 1. Introduction

Because of their outstanding mechanical properties and high temperature stability, silicon carbide (SiC) ceramics are potentially suitable materials for many different areas, such as aerospace, electronics, nuclear energy, and transportation [1,2,3,4]. The rapid development of aerospace, national defense, and nuclear energy has put forward urgent requirements for complex-shaped or large SiC ceramic components. However, due to the high abrasion resistance and hardness of SiC ceramics, their machinability is relatively poor. In this case, it is difficult to machine complex-shaped or large components by forging, extruding, and other plastic forming processes.

The joining technology is an effective way to realize complex-shaped or large SiC ceramic parts. A wide range of methods have been reported to join SiC ceramics, such as metallic braze-based joining [5], the use of MAX phases [6], diffusion joining [7], polymer-derived SiC joining [8], glass-ceramic joining joint [9], and Si–C reaction joining [10,11,12,13,14,15,16,17,18]. Among these methods, Si–C reaction joining is an attractive technique because the thermomechanical properties of the joint interlayer can be tailored to match those of the joining materials [16]. In addition, the Si–C reaction joining technology has many advantages. For example, the interlayer can be designed such that its thermomechanical properties match those of the joining materials, and joined specimens with high thermal stability can also be obtained [12,17]. This technology involves the infiltration of molten silicon into porous green bodies containing SiC and carbon powders, and the Si–C reaction occurs at 1450–1700 °C to form new SiC materials. The joint interlayer consists of three phases: new SiC, original SiC, and residual Si. M. Singh [11,12] investigated the effects of material composition and joining process parameters on the high-temperature mechanical properties of the joints formed by Si–C reaction joining. Li, S.B et al. [13] achieved the joining of reactively bonded SiC and recrystallized SiC using the reactive ligation method and found no changes in the steep gradient in the microstructure and properties of the interface between the joint and the substrate. Tian, W. B et al. [14] used TiB_2_–C cast films with different compositions and thicknesses as the joining layer material to join SiC ceramics via the infiltration of molten silicon at 1450 °C for obtaining dense TiB_2_–SiC joints. Luo [15] used SiC–C cast films with different compositions and thicknesses to join pressureless sintered SiC ceramics by the Si–C reaction joining method. However, most of the carbon precursors, such as graphite powder and petrol coke [10,11,12,13,14,15,16,17,18], were solid, which inevitably led to a complex and uncontrollable joining process. There are two main reasons for the poor joining performance: (1) it is difficult to obtain a uniformly distributed porous carbon green body; and (2) it is difficult to prepare a slurry with low viscosity and a high solid content. It has been reported that organic resins can be used instead of graphite powder as the carbon source for obtaining uniform porous carbon green bodies via polymerization-induced phase separation (PIPS) [19].

PIPS is a porous carbon preparation method that uses organic resins; in this method, the reactive monomer (RM) is phase-separated from the non-reactive component (NRC) after polymerization and then the NRC phase is removed by carbonization to form a porous structure [19]. Phenolic resin (PF) is an organic resin that can be used as a reactive monomer, and has the advantages of high carbon yields and excellent adhesion properties. For Si–C reaction bonding, sufficient infiltration of molten silicon at high temperatures is an important factor to obtain a stable joint interlayer, in which a more uniform and larger pore structure is more favorable for the infiltration of molten silicon. However, PF carbonization products have almost nano-scale pore structures, which are unfavorable for the infiltration of molten silicon at high temperatures. The key to control the pore structure obtained via the PIPS method is the separation of the resin phase and the solvent phase. Divalent metal ions can effectively increase the degree of polymerization of phenolic resins [20], which is beneficial for phase separation. Zhang Y et al. [21] have reported that Mg^2+^ can effectively enhance the solidification rate of PF.

In this study, the PF–MgCl_2_ system was used as the carbon precursor to replace the traditional solid carbon source, and the joining properties of SiC ceramics were investigated by the Si–C reaction joining method. The addition of MgCl_2_ could simply and effectively increase the pore structure after carbonization, making the infiltration of molten Si easier. The pore structures of the products obtained with and without MgCl_2_ addition were compared and studied. In addition, the effects of α-SiC powder as an inert filler on the microstructure and mechanical properties of the joints were investigated, and the joining mechanism was also discussed.

## 2. Experimental Methods and Procedures

### 2.1. Materials

Phenol-formaldehyde resin (PF) was purchased from FCP 15C, SIKA Tech, Lillesand, Norway. Ethylene glycol (EG, AR) and magnesium chloride (MgCl_2_, AR > 98.0%) were purchased from Aladdin Chemistry Co., Ltd., Shanghai, China. α-SiC powder (0.4 μm) was used as the inert filler, and it was purchased from FCP 15C, SIKA Tech, Lillesand, Norway. Polyvinylpyrrolidone (K-30, dispersant) was purchased from Shanghai Aladdin Co., Ltd. (Shanghai, China). Silicon carbide ceramics for joining were prepared by solid phase sintering at 2000–2150 °C, and the three-point bending strength was greater than 300 MPa. The surfaces of the silicon carbide ceramic samples used for joining were ground flat by a cylindrical grinder.

### 2.2. Preparation of Precursor Slurry

The preparation process of the resin mixture and its carbonization process are as follows: PF, EG, and MgCl_2_ were uniformly mixed by magnetic stirring at room temperature, cured at 90 °C for 6 h, and then maintained at 200 °C for 8 h. Finally, carbonization was carried out at 900 °C for 0.5 h under nitrogen atmosphere. The amount of MgCl_2_ added was 1% of the mass of the resin mixture.

The preparation process of the precursor slurry was similar to that of the resin mixture; the difference was the addition of the inert filler and the dispersant and the ball milling process. In this process, PF, solvent, MgCl_2_, polyvinylpyrrolidone (PVP), and inert filler were mixed by ball milling for 6 h. Furthermore, four slurries with different solid loadings were selected. The volume percentages of the solid particles in the slurries were 8%, 13%, 19%, and 26%, and the joined specimens were named W-1, W-2, W-3, and W-4, respectively.

### 2.3. Experimental Methods for Joining

**Figure 1:**
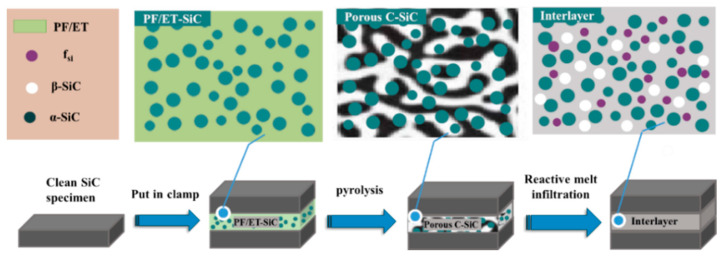
Schematic of preparation of SiC-SiC joining.

The dimensions of the SiC specimens used for joining were 6 mm × 30 mm × 40 mm, and those of the joint surface were 6 mm × 30 mm. In this study, the SiC specimens were ultrasonically cleaned in ethanol for 20 min before joining. The precursor slurry was uniformly coated between the surfaces of two SiC samples to form a sandwich structure, which was then loaded into a graphite mold and pyrolyzed at 900 °C. After pyrolysis and cooling, the graphite mold and pre-joining specimens were taken out from the sintering furnace. Finally, the pre-joining specimens were reacted with molten silicon at 1600 °C for 30 min in vacuum. A schematic diagram of the fabrication process of an SiC–SiC joint is shown in Figure 1.

### 2.4. Mechanical Properties Measurement

The apparent porosity, average pore size, and pore size distribution of the carbonized products were measured by a mercury porosimeter (AutoPore IV 9510, Ltd., McMurraytic Instruments Co., Norcross, GA, USA). The weight loss and weight loss rate during the polymerization of the resin mixture were examined using a thermogravimetric analyzer (TGA, SDTA85IE, METTLER TOLEDO, Zurich, Switzerland). The microstructure and backscattered electron (BSE) image of the joint were analyzed by scanning electron microscopy (SEM, S-4800, Hitachi, Tokyo, Japan). High-resolution images of the joint interface were obtained by transmission electron microscopy (TEM, JEM-2100F, JEOL Corporation, Tokyo, Japan). The flexural strength of the joined samples was tested by using an Instron 5566 test system, and the dimensions of the test sample were 3 mm × 4 mm × 36 mm.

## 3. Results and Discussion

### 3.1. Effect of MgCl_2_ on the Pore Structure

**Figure 2:**
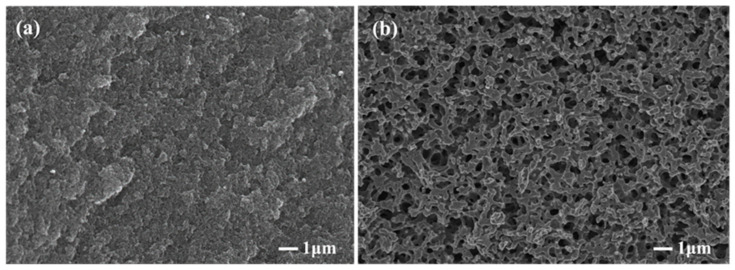
Effect of MgCl_2_ on the morphologies of porous carbons (**a**) without MgCl_2_; (**b**) with MgCl_2_.

Figure 2 shows the morphologies of the carbonized products of the resin mixture with and without MgCl_2_ addition. It is known that a porous structure is formed because of the removal of the solvent in the resin mixture and the carbonization of the phenolic resin. Compared with the microstructure of the carbonized product without MgCl_2_ addition (Figure 2a), a large number of three-dimensional interconnected pore structures could be observed in the carbonized product after the addition of MgCl_2_ (Figure 2b). The results reveal that the addition of MgCl_2_ has a beneficial effect on the formation of pores. The formation of three-dimensional interconnected pore structures is favorable for the infiltration of molten silicon during reaction sintering.

**Figure 3:**
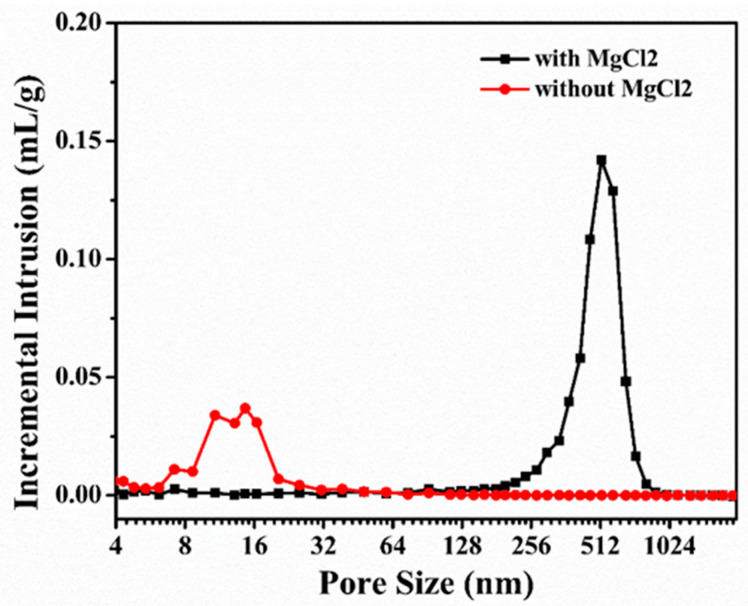
Pore size distributions deduced from mercury porosimetry.

In order to understand the specific effect of MgCl_2_ on the internal pore structure of porous carbon, the pore structure parameters were analyzed by a mercury porosimeter. The measured results were the average of the results of multiple tests, and they are displayed in Table 1. Compared with the average pore size of the carbonized products obtained without MgCl_2_ addition, the average pore size of the carbonized products obtained with MgCl_2_ addition increased from 14 ± 5 to 524 ± 21 nm; furthermore, the apparent porosity increased from 25.6 ± 1.1% to 51.68 ± 3.6%, and the bulk density decreased from 1.18 g·cm^−3^ to 0.78 g·cm^−3^. In addition, the pore size distribution of the carbonized product of the resin mixture measured using a mercury porosimeter is shown in Figure 3. The pore sizes of the carbonized product of the resin mixture without MgCl_2_ addition were mainly distributed between 10 and 25 nm. However, after the addition of MgCl_2_, the pore size distribution was significantly broadened, and the pore sizes ranged from 300 to 700 nm. It was observed that with the addition of MgCl_2_, the average pore size and apparent porosity of the carbonized product of the resin mixture significantly increased, and the results are consistent with the observations of the fracture surface morphology.

**Figure 4:**
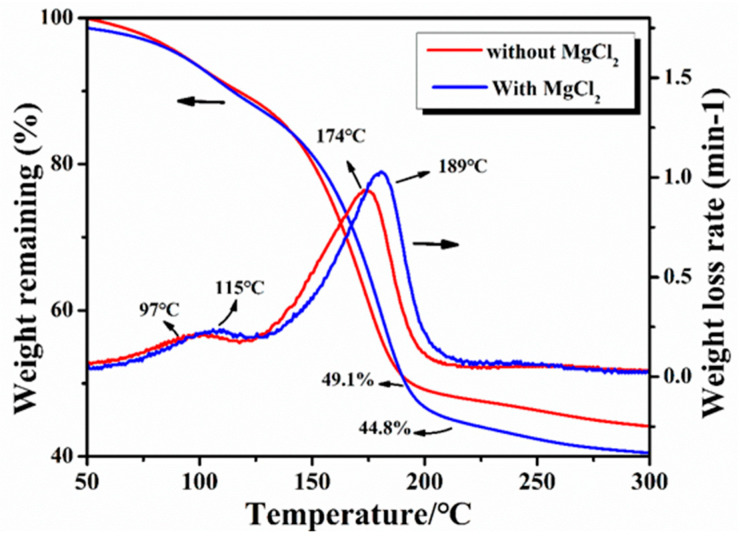
TG curves of the resin mixtures with and without MgCl_2_.

The changes in weight during the carbonization of the resin mixture were studied by thermogravimetry (TG). The results of the TG curves with and without the addition of MgCl_2_ are shown in Figure 4. It was observed that the mass retention of the resin mixture containing MgCl_2_ after curing at 200 °C was 44.8%, which indicates a decrease of about 5% compared with that of the resin mixture without MgCl_2_ (the mass retention was 49.1%). It can be seen from the TG weight loss rate curve that the resin mixture without MgCl_2_ exhibits two peaks at 97 °C and 174 °C, corresponding to the weight loss caused by the volatilization of water molecules and ethylene glycol. However, with the addition of MgCl_2_, the temperatures of the maximum weight loss rates for water molecules and ethylene glycol increased slightly: 115 °C and 189 °C, respectively. Accordingly, a change in the peak position of the weight loss rate was observed because the complex reaction of MgCl_2_ with the resin increased the degree of curing, making volatilization more difficult.

In the phenolic structure of phenolic resins, the para position is the most reactive position, whereas the ortho position has lower reactivity [20]. Therefore, during the reaction process, when the -CH_2_OH group was polymerized at the ortho position of the phenol, its reactivity gradually decreased. However, after the addition of MgCl_2_, the positioning effect of Mg^2+^ could promote the ortho substitution of the -CH_2_OH group [20], due to which the ortho position also exhibited high reactivity; this increased the efficiency of the whole reaction and finally, a higher degree of polymerization was obtained. The increase in the degree of polymerization of PF accelerated the separation of the PF phase and the solvent phase. The increase in the degree of the separation of the two phases favored the formation of a larger solvent-rich phase, which volatilized at high temperatures to form larger pore sizes. This is an important reason for adding MgCl_2_. The increase in the degree of polymerization of PF enhances the resistance of a resin to shrinkage during pyrolysis, which is beneficial to obtain higher apparent porosity [22].

### 3.2. Microstructure of the Precursor Slurry after Pyrolysis

**Figure 5:**
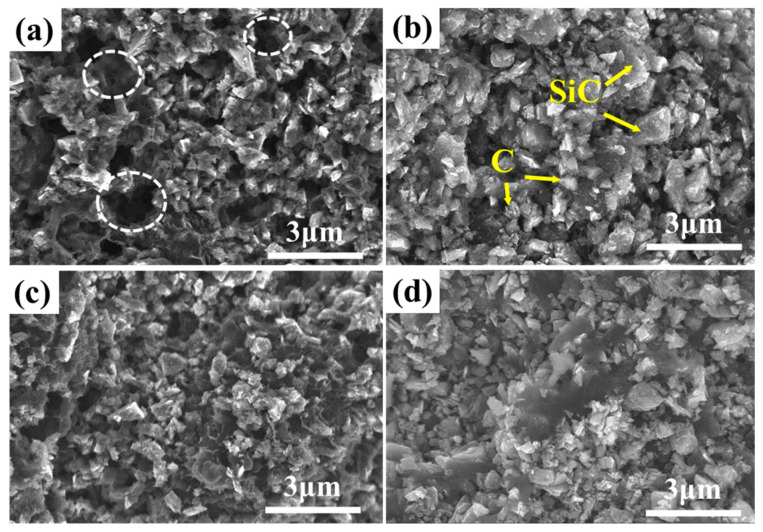
Microstructure of the phenolic resin pyrolysis with different solid loading: (**a**) 8%; (**b**) 13%; (**c**) 19%; (**d**) 26%.

Pure resins are not ideal for the high-temperature joining of SiC ceramic materials mainly due to the large volume shrinkage of phenolic resins after pyrolysis. Due to volume shrinkage, the pre-bonding layer cannot provide the porous structure required for high-temperature silicon infiltration. To reduce the volume shrinkage due to PF pyrolysis, inert fillers were added to the resin mixture. After pyrolysis, silicon carbide/carbon (SiC/C) porous green bodies were obtained, and their microstructures and properties were systematically investigated. Figure 5 shows the microstructures of the fractures of SiC/C porous preforms with different SiC contents. It can be seen from the figure that the carbonized product of the precursor slurry mainly contains SiC particles and carbon formed by the carbonization of PF. Moreover, it can also be observed that silicon carbide and carbon together constitute the skeleton of the carbonized product of the precursor slurry, and the pore structure presents a three-dimensional network. Meanwhile, no agglomeration occurred among the particles, indicating that the dispersant and the ball milling process could achieve uniform dispersion of micron-sized SiC particles in the phenolic resin/alcohol premix.

In addition, when the solid content was 8% (Figure 5a), the carbon particles were connected to each other, and the SiC particles were wrapped and connected together to form a skeleton structure; however, defects such as holes still tended to appear. After reactive infiltration, these holes were easily filled with molten silicon, forming large pieces of free silicon; this affected the joining performance. However, with the increase in the solid loading (Figure 5b–d), the microstructure of the carbonized product of the precursor slurry gradually tended to be uniform. Importantly, the SiC particles could also be uniformly distributed in the continuous phase matrix composed of carbon particles, and the skeleton structure was mainly composed of carbon–SiC particles, which in turn reduced the appearance of macropores. Hence, a uniformly distributed porous structure can be obtained with the addition of inert fillers, which is favorable for the infiltration of molten silicon at high temperatures.

### 3.3. Microstructure of the Joined Specimens

**Figure 6:**
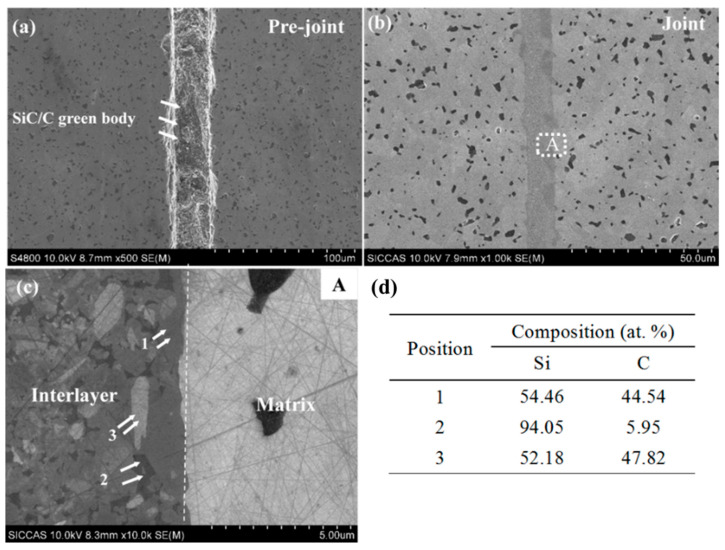
Microstructure of the joint of W-4: (**a**) Pre-joint; (**b**) joint; (**c**) region A in (**b**); (**d**) the element compositions of the regions marked in (**c**).

The microstructures and EDS results of the pre-joining and joining specimens were investigated (Figure 6). The joint showed a dense microstructure; moreover, no cracks were observed and no pores were present at the interlayer and the interface. In the EDS results, the light gray area (Figure 6c,d(1,3)) and the dark gray area (Figure 6c,d(2)) in the interlayer represent the new SiC phase and the residual Si phase (fSi), respectively, indicating the uniform distribution of silicon carbide and residual silicon in the layer. Moreover, it can be seen from Figure 6 that SiC–SiC and Si–SiC are mainly observed at the joint interface, indicating that the pyrolyzed carbon can completely react with molten silicon at 1600 °C after siliconizing to form a β-SiC phase.

**Figure 7:**
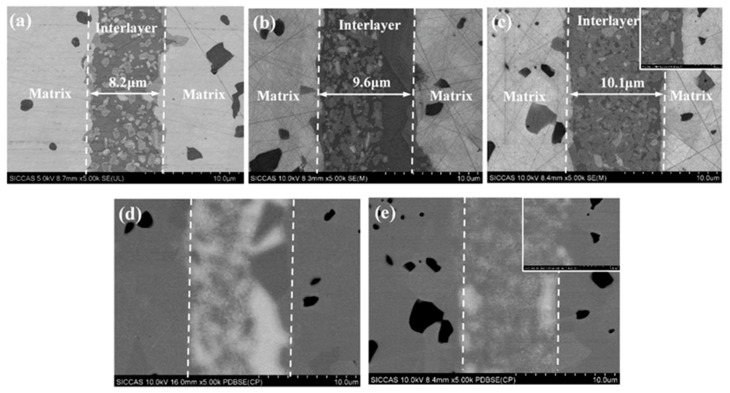
Microstructure and EDS of the joint with different solid loading: (**a**) 13%; (**b**) 19%; (**c**) 26; (**d**) the PDBSE of (**b**); (**e**) the PDBSE of (**c**).

The microstructures of the joined specimens with different solid loadings were also investigated (Figure 7). Specifically, the joint thicknesses of W-2 (Figure 7a), W-3 (Figure 7b), and W-4 (Figure 7c) were approximately 7.8 μm, 9.6 μm, and 10.1 μm, respectively. It is well-known that the weakest part of a joint, such as free silicon, determines the reliability of the joined specimen [23,24]. Therefore, in order to form joined specimens with strong interfacial connection, the interlayer should have a uniform microstructure and it should be free of defects. The BSE images show that the content of the residual silicon phase (Figure 7d,e, white area) in the interlayer of W-4 (Figure 7e) is lower than that in the interlayer of W-3 (Figure 7d). This indicates that the joined samples with higher solid contents have a more uniform microstructure and a lower content of free silicon, which are beneficial to the improvement in the joining performance.

**Figure 8:**
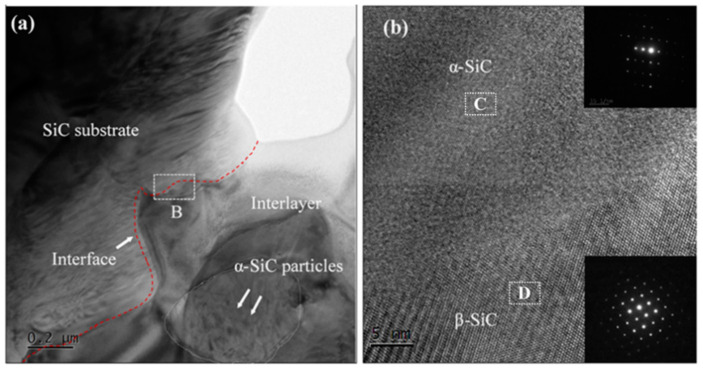
TEM image and the corresponding HRTEM of (**a**) SiC-SiC joint, (**b**) B regions of (**a**).

Figure 8 shows the TEM image of the joint and a high-resolution image at the interface. Figure 8a indicates that the new SiC formed by reaction bonding is distributed in the region between the SiC substrate and the added SiC particles in the interlayer. Figure 8b shows that the interface between the joint and the SiC substrate is clear without a transition layer. This indicates that the new SiC formed on the substrate surface was stably attached to the SiC substrate. From the electron diffraction images in Figure 8b, it can be seen that there are α-SiC and β-SiC phases in the C and D regions, respectively. The C regions is SiC substate of Figure 8a. The D regions is interlayer of Figure 8a.This indicates that the newly formed SiC was mainly the β-SiC phase. The joining process is as follows: when molten silicon was flowing sufficiently, carbon dissolved in the molten silicon, forming a carbon activity gradient and C-Si pairs. Then, the C–Si pairs diffused to cooler locations (i.e., the SiC substrate or the added α-SiC particles) and were supersaturated, and a new SiC layer was formed on the surface of the SiC substrate or on the added SiC particles. This was consistent with the dissolution-precipitation mechanism [25,26]. The newly formed β-SiC phase, the substrate, and the SiC particles in the joint interlayer were firmly joined, forming a stable joint.

### 3.4. Mechanical Properties of the Joined Specimens

**Figure 9:**
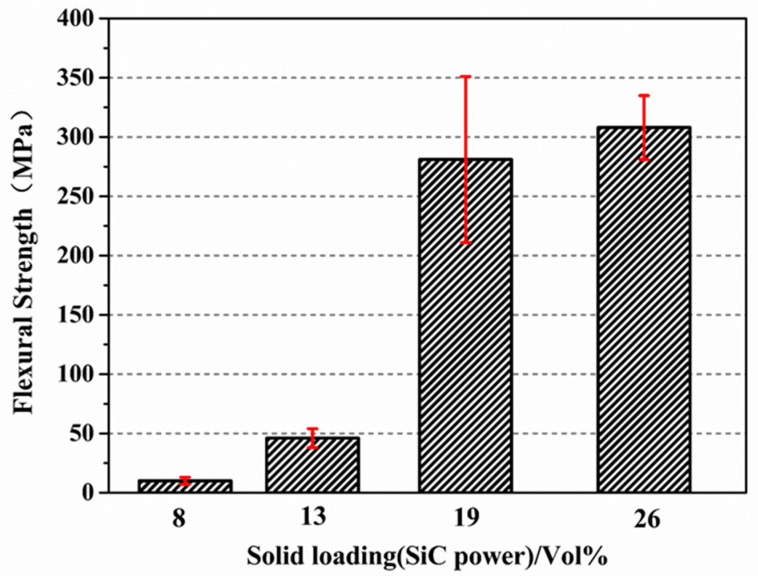
Flexural strengths of the joining specimens at room temperature with different solid loading (8%, 13%, 19%, 26%).

The results for the flexural strengths of the joined specimens at room temperature with different solid loadings (8 vol%, 13 vol%, 19 vol%, and 26 vol%) are shown in Figure 9. The flexural strength of the joined specimens increased from 10 ± 3 MPa, 46 ± 8 MPa to 280 ± 70 MPa as the solid loading content increased from 8%, 13% to 19%, respectively. When the solid loading content was 26%, the flexural strength reached 308 ± 27 MPa. It can be seen from Figure 9 that the addition of SiC has a significant influence on the joining performance. The increase in the flexural strength was mainly due to the following three reasons: first, the addition of SiC powder reduced the volume shrinkage after PF pyrolysis at high temperatures. Second, as the inert filler (α-SiC powder) content increased, the pore distribution and SiC/C distribution in the green body after PF pyrolysis became uniform, which contributed to the infiltration of molten Si at high-temperature [27]. Finally, the content of the residual silicon phase in the interlayer decreased gradually with the increase in the content of the inert filler, and the SiC and Si phases tend to be homogeneously distributed.

## 4. Conclusions

In this study, SiC–SiC joints were produced by a Si–C reaction bonding method using the phenolic resin (PF)–MgCl_2_ system as the carbon precursor. This approach was adopted with the aim to increase the pore size after carbonization, thus making the infiltration of molten Si easier. Both the microstructure of the joined specimens and the influence of SiC powder as the inert filler were evaluated. It was demonstrated that the addition of MgCl_2_ considerably increased the average pore size of the carbonized product from 14 ± 5 to 524 ± 21 nm. The flexural strength of the joined specimens increased with the increase in the solid loading of the inert filler. The flexural strength was the highest, i.e., 308 ± 27 MPa when the solid loading of the inert filler was 26%. Overall, stable joining of silicon carbide ceramics was achieved by the proposed method, which has significance for realizing the preparation of complex-shaped or large silicon carbide ceramic parts.

## Figures and Tables

**Table 1:** Effect of MgCl_2_ on the pore structure parameters of carbonized product.

Sample	Apparent Porosity (%)	Bulk Density (g·cm^−3^)	Pore Volume/cm^3^·g^−1^	Average Pore Size (nm)
With MgCl_2_	51.68 ± 3.6	0.78 ± 0.03	0.66 ± 0.05	523 ± 21
Without MgCl_2_	25.6 ± 1.1	1.18 ± 0.08	0.21 ± 0.07	14 ± 5

## Data Availability

The data presented in this study are available on request from the corresponding author.
